# 66942 Metabolomic endotype of bioenergetic dysfunction predicts mortality in critically ill patients with acute respiratory failure

**DOI:** 10.1017/cts.2021.669

**Published:** 2021-03-30

**Authors:** Raymond J. Langley, Marie M. Migaud, D. Clark Files, Peter Morris

**Affiliations:** 1 University of South Alabama; 2 Wake Forest Baptist Medical Center; 3 University of Kentucky Medical Center

## Abstract

ABSTRACT IMPACT: The pathophysiologic features of a metabolomic endotype that predicts patient outcomes due to sepsis have the potential to direct new therapies that target immune dysregulation and bioenergetic insufficiency. OBJECTIVES/GOALS: Acute respiratory failure (ARF) requiring mechanical ventilation is a frequent complication of sepsis and other disorders. It is associated with high morbidity and mortality. Despite its severity and prevalence, little is known about metabolic and bioenergetic changes that accompanying ARF. METHODS/STUDY POPULATION: In this study, semiquantitative and quantitative ultrahigh performance liquid chromatography mass spectrometry (UHPLC MS) analysis was performed on patient serum collected from the Trial with Acute Respiratory failure patients: evaluation of Global Exercise Therapies (TARGET). Serum from survivors (n=15) and nonsurvivors (n=15) was collected at day 1 and day 3 after admission to the medical intensive care unit as well as at discharge in survivors. Pathway analysis of the biochemical changes was performed to determine whether the disruption in specific metabolic pathways can identify the bioenergetic and metabolomic profile of these patients. RESULTS/ANTICIPATED RESULTS: Significant metabolomic differences were related to biosynthetic intermediates of redox cofactors nicotinamide adenine dinucleotide (NAD) and NAD phosphate (NADP), increased acyl-carnitines, and decreased acyl-glycerophosphocholines in nonsurvivors compared to survivors. The metabolites associated with poor outcomes are substrates of enzymatic processes dependent on NAD(P), while the abundance of NAD cofactors rely on the bioavailability of dietary vitamins B1, B2 and B6. Changes in the efficiency of the nicotinamide-derived cofactors’ biosynthetic pathways also associate with an alteration of the glutathione-dependent drug metabolism as characterized by the substantial differences observed in the acetaminophen metabolome. DISCUSSION/SIGNIFICANCE OF FINDINGS: This metabolomic endotype represents a previously unappreciated association between severity of outcomes and micronutrient deficiency, thus pointing to new pharmacologic targets and highlighting the need for nutritional remediation upon hospitalization to improve patient outcomes due to ARF.

